# *Sprouty* gene dosage influences temporal-spatial dynamics of primary enamel knot formation

**DOI:** 10.1186/s12861-015-0070-0

**Published:** 2015-04-22

**Authors:** Katerina Lochovska, Renata Peterkova, Zuzana Pavlikova, Maria Hovorakova

**Affiliations:** Institute of Experimental Medicine, Academy of Sciences of the Czech Republic, Prague, Czech Republic; Department of Anthropology and Human Genetics, Faculty of Science, Charles University, Prague, Czech Republic

**Keywords:** Enamel knot, Tooth development, Mouse molar, *Sprouty* genes, Sonic hedgehog, Cre-loxP system, Supernumerary tooth

## Abstract

**Background:**

The mouse embryonic mandible comprises two types of tooth primordia in the cheek region: progressive tooth primordia of prospective functional teeth and rudimentary tooth primordia in premolar region – MS and R2. Mice lacking *Sprouty* genes develop supernumerary tooth in front of the lower M1 (first molar) primordium during embryogenesis. We focused on temporal-spatial dynamics of *Sonic Hedgehog* expression as a marker of early odontogenesis during supernumerary tooth development.

**Results:**

Using mouse embryos with different dosages of *Spry2* and *Spry4* genes, we showed that during the normal development of M1 in the mandible the sooner appearing *Shh* signaling domain of the R2 bud transiently coexisted with the later appearing *Shh* expression domain in the early M1 primordium. Both domains subsequently fused together to form the typical signaling center representing primary enamel knot (pEK) of M1 germ at embryonic day (E) 14.5. However, in embryos with lower *Spry2*;*Spry4* gene dosages, we observed a non-fusion of original R2 and M1 *Shh* signaling domains with consequent formation of a supernumerary tooth primordium from the isolated R2 bud.

**Conclusions:**

Our results bring new insight to the development of the first lower molar of mouse embryos and define simple tooth unit capable of individual development, as well as determine its influence on normal and abnormal development of the tooth row which reflect evolutionarily conserved tooth pattern. Our findings contribute significantly to existing knowledge about supernumerary tooth formation.

## Background

Similarly to the development of other organs, tooth morphogenesis is a complex multifactorial process involving fundamental morphogenetic mechanisms (proliferation, apoptosis, integration and migration of cells), which are controlled by interactions between epithelium and mesenchyme.

Mouse adult dentition comprises one incisor, which is separated from three molars by a toothless diastema in each jaw quadrant. Although adult mouse diastema does not contain teeth, there are tooth rudiments transiently apparent in the upper and lower embryonic diastema [1]. In mouse embryonic lower diastema two large rudimentary tooth buds (called MS and R2) appear during embryonic development [2] and they have been associated with premolar teeth lost during evolution [1,3]. The larger posterior rudiment (R2) is incorporated into the rising cap of the functional first molar (M1) [2,4].

Enamel knots (EKs) are transient signaling centers comprising non-dividing cell population in dental epithelium [5-7]. EKs are important structures initiating and regulating tooth shape and determining the number of tooth cusps [5-7]. They were considered to be the main regulator of tooth development [7]. It has been discovered that the primary enamel knot (pEK) controls morphogenesis in the first molar germ in mouse [6] and that *Sonic Hedgehog* (*Shh*) is expressed there [8]. During early stages of odontogenesis, *Shh* expression is limited to the epithelial cells and it is considered as an early marker of odontogenesis [9,10]. The *Shh* stimulates proliferation of epithelial cells in areas of early tooth development [11]. Moreover, *Shh* is also expressed in the signaling centers of the MS and R2 rudiments [4]. In the present study, we showed that M1 pEK in its accepted meaning (as a signaling center of a tooth) arises as late as after the fusion of the original R2 and early M1 *Shh* signaling domains.

A supernumerary tooth in front of molars was found in adult *Spry2* or *Spry4* knock out mice [12,13]. The premolar rudiments MS and R2 have been assumed to take a part in the formation of supernumerary tooth in front of M1 [4,14].

Gene dosage is an issue that is far from being explored. Research is carried out in particular at the level of chromosomes mainly dealing with chromosomal or segmental aneuploidy (e.g. [15-17]). Transgenic mice enable to study the dose of one or two selected genes. Using *Wise*-null mice, it has been proven that phenotypes of tooth (incisors and molars) number depends on varying doses of the *Lrp5* and *Lrp6* co-receptor genes [18]. Similarly, it has been proven that doses of the *Fgfr1* and *Fgfr2* genes affect formation of diastemal tooth in *Sprouty*2 deficient mice [13]. Using *Sprouty2;Sprouty4* (*Spry*) transgenic mice, it has been found that number of incisors may be influenced by the level of activity of a single signal transduction pathway [19].

However, the information about relationship of *Spry2* and *Spry4* gene dosages and early tooth development in the cheek region is missing. We presented here that a lack of *Sprouty*2 and *Sprouty*4 alleles influences the dynamics of the *Shh* expression in the lower jaw of *Spry*2;*Spry*4 mutant mice, as well as the development of the R2 rudiment and the M1. This influence increased with the decreased number of functional *Sprouty* alleles. We proved that with decreased dosages of *Sprouty* genes, the signaling center of R2 rudiment did not participate in the M1 formation and it stayed separate becoming a signaling center of the supernumerary tooth primordium in front of M1 anlage.

## Results

### Dynamics of *Shh* expression influenced by *Sprouty* gene dosages

For all nine possible genotypes (Table 1), the shape of dental epithelium and a presence and pattern of *Shh* expression domains were evaluated during the tooth development in the lower jaw from E11.5 till 16.5 (embryos with body-weight between 30–800 mg). The sequential occurrence of the *Shh* expression was determined in the rudiment MS, rudiment R2 and M1 anlage in mutants and compared to controls (*Spry2*+/+;*Spry4*+/+).

**Table 1:** **Numbers of processed samples for single genotypes**

**Genotypes**	**Amount of samples**
S2+/+;S4+/+	52
S2+/+;S4+/−	107
S2+/+;S4−/−	70
S2+/−;S4+/+	108
S2+/−;S4+/−	98
S2+/−;S4−/−	90
S2−/−;S4+/+	43
S2−/−;S4+/−	51
S2−/−;S4−/−	10
**in total**	**629**

Lower number of *Spry2*−/−;*Spry4*−/− mutant embryos compared to other genotypes could be caused by their early prenatal lethality that might be related to a high incidence of additional developmental defects [48].

### Higher *Spry*2;*Spry*4 gene dosages

**Figure 1:**
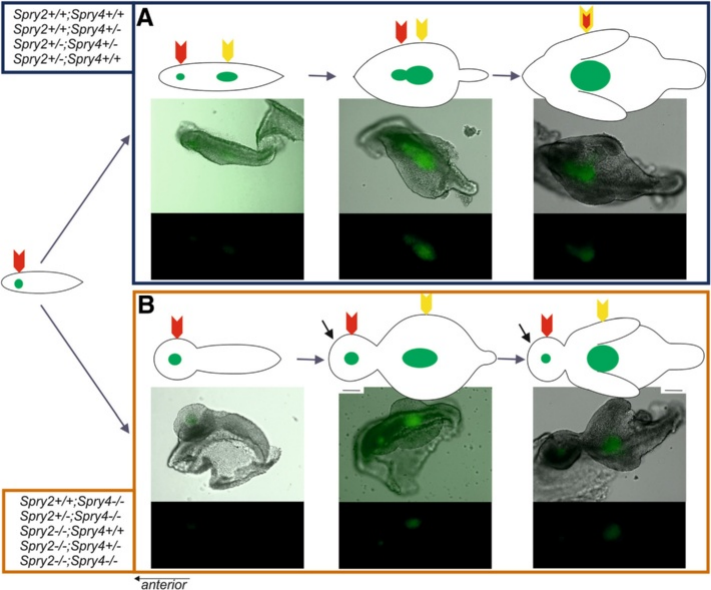
Physiological and pathological tooth formation in dissociated epithelia of mouse lower cheek region. **(A)** In samples with higher *Spry*2;*Spry*4 gene dosages (*Spry2*+/+;*Spry4*+/+, *Spry2*+/+;*Spry4*+/−, *Spry2*+/−; *Spry4*+/−, *Spry2*+/−; *Spry4*+/+), R2 *Shh* signaling domain (red arrow) merges with early M1 *Shh* signaling domain (yellow arrow) to form a typical elongated pEK (double yellow-red arrow) of the germ of M1. **(B)** In samples with lower *Spry*2;*Spry*4 gene dosages (Spry2+/+; Spry4−/−, Spry2+/−; Spry4−/−, Spry2−/−; Spry4+/+, Spry2−/−; Spry4+/−, Spry2−/−; Spry4−/−), R2 signaling domain (red arrow) persists in front of M1 signaling domain (yellow arrow) and became a signaling center of supernumerary tooth primordium (black arrow). Anterior direction is on the left side (the scale bar is 100 μm).

A group with higher *Spry2*;*Spry4* gene dosages comprised the control genotype (*Spry2*+/+;*Spry4*+/+) and the genotypes with one mutant allele of one or both *Spry2* and/or *Spry4* genes (*Spry2*+/−;*Spry4*+/+, *Spry2*+/+;*Spry4*+/−, *Spry2*+/−;*Spry4*+/−). The samples showed similarities in *Shh* expression dynamics and in shape (Figure 1A) of dental epithelia during development.

**Figure 2:**
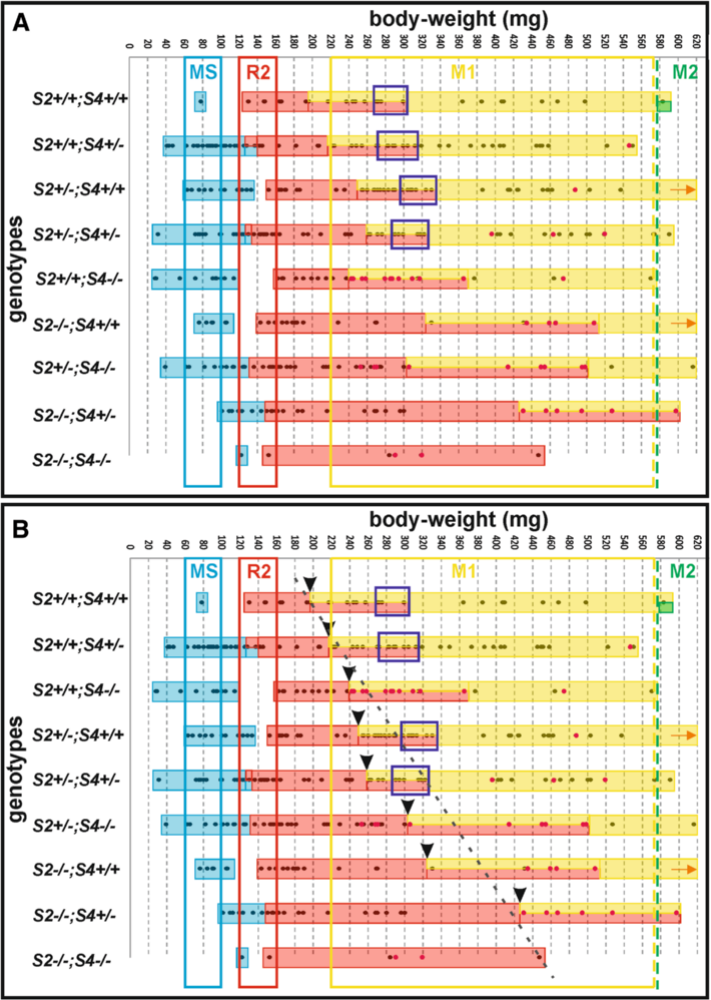
*Shh* expression in three distinct signaling domains (MS, R2, M1) in *Spry2;Spry4* samples. *Shh* expression in three distinct signaling domains: MS (blue bar), R2 (red bar) and M1 (yellow bar), was sequentially detected in the cheek region of embryonic mandibles in nine various *Spry*2;*Spry*4 mouse genotypes according to embryonic body weights. **(A)** The supernumerary tooth formation is dependent on decreasing dosages of *Spry*2 and *Spry*4 genes. The samples with higher *Spry*2;*Spry*4 gene dosages showed merging (purple frames) of R2 and early M1 *Shh* signaling domains into one expression domain (pEK) located in the center of M1 germ. In contrast, samples with lower *Spry*2;*Spry*4 gene dosages did not evince any merging. Instead of this, they showed a supernumerary tooth primordium which arose from persisting R2 with its own *Shh* signaling domain. **(B)** The prolongation of *Shh* expression was strengthened with decreasing *Spry*2 gene dosage. The prolongation of *Shh* expression in preceding signaling domain implies later start of subsequent expression domain. Black arrowheads represent the moment where the early M1 *Shh* signaling domain appeared and it co-existed transiently with the persisting *Shh* expression in R2. Dashed black line suggests the trend of prolongation of *Shh* expression in R2 rudiment according to decreasing *Spry2* gene dosage. The colored frames (blue, red and yellow) represent referential presence of signaling domains of MS (blue), R2 (red) and M1 (yellow) observed in WT mice [4]. The green dashed line is a reference line representing M2 (second molar) signaling center appearance in control genotype (*Spry2+/+;Spry4+/+*). The density of harvested embryos is shown by dots in bars. Pink dots represent embryos with supernumerary tooth germ formation. Orange arrow determines continuation of yellow bar given by harvested material with higher body-weight out of the graph field. Gaps between colored bars mean absence of material.

The first *Shh* expression domain localized in MS rudiment was detected from E11.5 until 13.5 (embryos with body-weight until approximately 130 mg). It was followed by R2 signaling domain from E13.5 until 14.7 (body-weigh in range 130–320 mg). In two genotypes of this group (*Spry2*+/+;*Spry4*+/− and *Spry2*+/−;*Spry4*+/−) we noticed synchronous presence of two separate signaling centers corresponding to MS and R2 at E13.5 (body-weight approximately 130 mg), but only for a short time and both signaling domains were very weak. From E14.3 until 14.5 (body-weight in range 220–270 mg) the dissociated epithelia showed two isolated *Shh* expression domains in R2 and M1 primordia. Between E14.5 and 14.7 (body-weight in range 270–320 mg) the fusion of these two (originally isolated) domains was obvious (Figure 1A; Figure 2A). After that, only one single *Shh* domain persisted corresponding to the pEK of M1 from E14.7 until 16.5 (body-weight in range 320–580 mg). All the above-mentioned chronological parameters were summarized for the four genotypes with higher dosages of *Spry*2 and *Spry*4 genes (for details see in Figure 2). Interestingly, the development in general was slightly delayed compared to control mice (*Spry2*+/+; *Spry4*+/+) in all transgenic specimens (Figure 2B).

### Lower *Spry*2;*Spry*4 gene dosages

**Figure 3:**
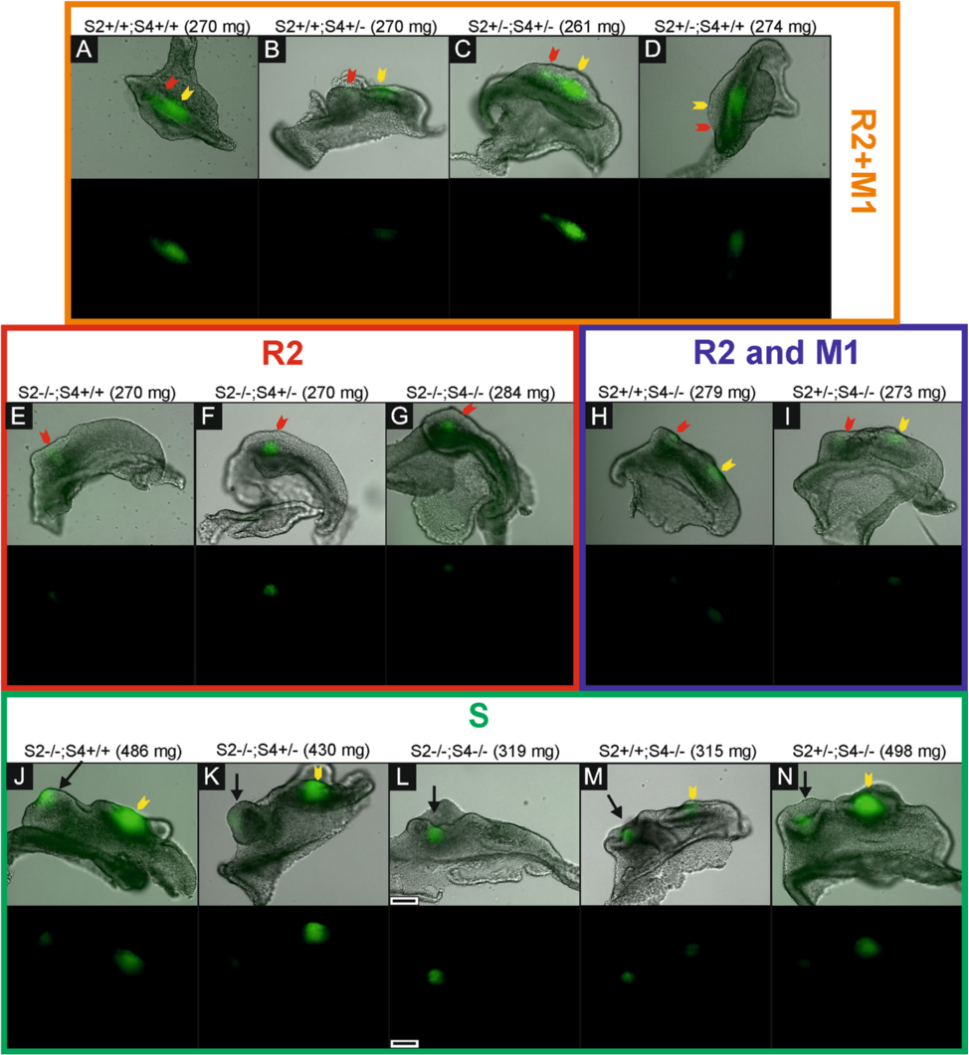
Differences in the tooth development according to the dosages of *Spry*2 and *Spry*4 genes. Bright field images of dissociated epithelia combined with GFP visualization of *Shh* expression (upper lines) supplemented with GFP only panels (lower lines) show distinct patterns of *Shh* signaling domains during tooth development in mouse mandible. **(A-D)** In specimens with similar body weights (around 270 mg), it means at the same level of the development, the dissociated epithelia in the samples with higher *Spry*2;*Spry*4 gene dosages (in orange rectangle) already show merging of R2 (red arrow) and early M1 (yellow arrow) *Shh* signaling domains preceding the pEK formation of prospective functional M1. In contrast, samples with lower *Spry*2;*Spry*4 gene dosages exhibit only single R2 signaling domain (**E**-**G** in red rectangle) or separate *Shh* signaling domains of R2 (red arrow) and M1 (yellow arrow) (**H**-**I** in blue rectangle). **(J-N)** The persisting R2 rudiment in the genotypes with lower *Spry*2;*Spry*4 gene dosages gives rise to a supernumerary tooth primordium (S, black arrow) in front of M1 at later stages (in green rectangle). Anterior direction is on the left side (the scale bar is 100 μm).

The further five genotypes formed a group with lower *Spry2*;*Spry4* gene dosages, where both alleles of at least one of *Spry2* or *Spry4* genes are mutant (*Spry2−/−*;*Spry4*+/+, *Spry2*+/+;*Spry4*−/−, *Spry2*+/−;*Spry4*−/−, *Spry2*−/−;*Spry4*+/−, *Spry2*−/−;*Spry4*−/−). The distinction was found in temporal-spatial distribution of *Shh* signaling domains compared to the samples with higher *Spry2*;*Spry4* dosages during development (Figure 1B; Figure 2B). *Shh* was expressed in the signaling centers of R2 and M1 similarly to the specimens with higher *Spry2*;*Spry4* gene dosages. However, there was a prolongation of the *Shh* expression in R2 resulting in delayed start of *Shh* expression in M1 compared to control (*Spry2*+/+; *Spry4*+/+). This became more evident with decreasing dosages of *Spry2* genes (Figure 2B). Moreover, the *Shh* expression domains of the R2 and M1 never merged together (compare Figure 1 and Figure 3). This non-fusion resulted in the formation of a supernumerary tooth primordium from the autonomous development of the unmerged R2 rudiment anteriorly to M1 tooth germ.

### Supernumerary tooth formation

We evaluated all samples for presence of the supernumerary tooth primordium (criterion see in Methods). The supernumerary tooth primordium occurred in all evaluated genotypes (except of controls), but its appearance varied depending on the *Sprouty* gene dosages. With decreasing *Spry2*;*Spry4* gene dosages, the incidence of the supernumerary tooth primordium increased. The development of dental epithelium in the cheek region was different between the embryos with higher and lower *Spry* gene dosages (without and with the supernumerary tooth formation, respectively). The genotypes with higher *Spry*2;*Spry*4 gene dosages (*Spry2*+/+;*Spry4*+/+; *Spry2*+/−;*Spry4*+/+; *Spry2*+/+;*Spry4*+/−; *Spry2*+/−;*Spry4*+/−) showed normal progress of tooth development in the cheek region (Figure 1A). In contrast, in the genotypes with lower *Spry*2;*Spry*4 gene dosages (*Spry2*−/−;*Spry4*+/+; *Spry2*+/+;*Spry4*−/−; *Spry2*−/−;*Spry4*+/−; *Spry2*+/−;*Spry4*−/−; *Spry2*−/−;*Spry4*−/−) only the early shape of dental epithelium exhibiting MS expression domain was similar to control samples (if we do not take into account the embryonic stage of development). However, when the MS signal disappeared, the anterior part of the epithelium enlarged and reached a “button”-shape (from aerial view Figure 1B). The posterior part of dental epithelium was narrow with a straight tail. In the middle of the enlarged anterior part, a rounded *Shh* signaling domain was located corresponding to the R2 signaling domain and persisting until later stages as a signaling center in the separate supernumerary tooth primordium. During the subsequent development, the posterior part of the dental epithelium in the cheek region was extended forming the M1 cap with an elliptical *Shh* expression domain while the anterior part did not change. Then, both parts developed into two separate tooth primordia: supernumerary tooth germ and M1 germ (Figure 1B).

### The tracing of the fate of cells expressing *Shh* in supernumerary tooth primordium

To investigate the relationship of the R2 *Shh* signaling domain and supernumerary tooth primordium formation in mouse embryonic mandible, we generated *Spry4*−/−*Shh*ERCre mouse strain and *Spry4*−/−*Rosa26-LacZ* line. These mice exhibited dental phenotype of *Spry4* deficient mice and allowed preparing tamoxifen inducible Cre-loxP system for tracing of the fate of cells expressing *Shh* in R2 rudiment in mice with supernumerary tooth formation.

**Figure 4:**
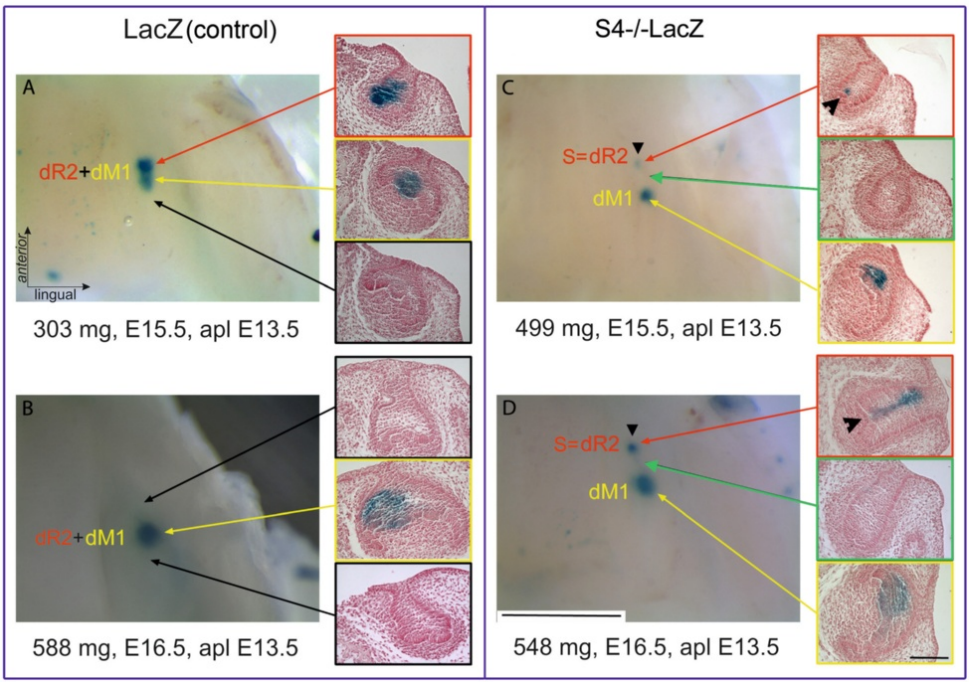
The tracing of cells originally expressing *Shh* in tooth primordia visualized by X-gal staining. Left – whole mount staining of the lower jaw, right - corresponding histological sections. The *Shh* expressing cells and their descendants in the R2 rudiment and M1 germ are compared during supernumerary tooth formation in *Spry4-/-* mutants and control mice. The tamoxifen dose was administrated into pregnant female mice LacZ **(A, B)** and *Spry4-/-*LacZ **(C, D)** at E13.5, because *Shh* is expressed in R2 rudiment in this time. In control embryos, merging of two labelled descendant cell populations in areas of original R2 (dR2, red arrow) and M1 (dM1, yellow arrow) at E15.5 was clearly detectable **(A)** leading to a single area of blue cells in M1 germ at E16.5 **(B)**. In contrast, *Spry4-/-*LacZ embryos showed two isolated areas of blue cells indicating descendant cell populations of original R2 (dR2) and M1 (dM1) at E15.5 **(C)** as well as at E16.5 **(D)**. The area between descendant original R2 and M1 showed no *Shh* expression (green arrow). The anterior area of blue cells was located in the center of developing supernumerary tooth primordium (S, black arrowhead) originating in the R2 rudiment. Black arrows indicate the areas of tooth germs shown on histology without *Shh* expression. The scale bar indicates 2 mm on whole mount, or 100 μm on slices.

The tamoxifen dose was administrated into pregnant female mouse at E13.5, it means before the fusion of original R2 and early M1 *Shh* signaling domain under physiological conditions. Embryos were harvested at E15.5 and 16.5, when only M1 pEK was present in controls (Figure 4A, B). X-gal staining visualized the cells expressing *Shh* (blue cells) from the time of tamoxifen injection and all their descendants until harvesting of embryos. We found two distinct and separate blue areas in the cheek region of *Spry*4 deficient mouse mandibles. Histological sections showed that the posterior area of the blue cells was located in the M1 primordium, and the anterior area of the blue cells was located in the center of the supernumerary tooth cap (Figure 4C, D). This proved the persistence of cells of the originally separate R2 *Shh* signaling domain in the center of the supernumerary tooth primordium. These blue-labelled cells of the R2 were separated by a negative zone from the labelled cells of the M1, they did not mix with the cells expressing *Shh* and their descendants in the early M1. It implies that the fusion of original R2 *Shh* expression domain with early M1 *Shh* domain was absent and the formation of M1 pEK was disrupted. This resulted in the pathological development of the tooth row. The group of cells expressing *Shh* in original R2 rudiment and their descendants remained separate as the proper signaling center of the supernumerary tooth.

## Discussion

The presence of dental rudiments in the antemolar space of mouse embryonic jaws has been previously demonstrated on the basis of a combination of histology, morphometry and 3D reconstructions [2,20-22]. It has been shown that the *Shh* expression domains of individual structures (MS, R2, M1) appear sequentially in the anterior-posterior series in the cheek region of the mouse embryonic mandible [4].

### Formation of primary enamel knot

It is generally accepted that pEK starts to appear at the tip of tooth bud (E13) and is clearly detectable at cap stage (E14) [5,23,24]. According to our results, there are two isolated *Shh* expression domains appearing in R2 and M1 primordia in the cheek region of mandible that have been already detected in WT mice [4]. The sensitive detection using isolated epithelia in the present study clearly documented the transient synchronous co-expression of *Shh* in the R2 rudiment and original early M1 and their following fusion between E14.5 and 14.7 forming one composite *Shh* expression domain – called pEK in the center of prospective functional M1. Based on this new insight, the original early *Shh* expression in M1 should not be identified as pEK, since the typical oblong pEK in M1 germ forms as late as after the fusion of the former *Shh* signaling centers in the R2 rudiment and original M1 germ.

### Non-fusion of R2 and M1 *Shh* expression domains results in supernumerary primordium formation

Molecular and genetic studies over the last twenty years have shown that the development of dentition is a dynamic and very complex process controlled by numerous signaling pathways. These processes determine the appropriate shape, number and pattern of teeth [25]. It is obvious that errors can occur during such complicated process, resulting in formation of oral pathologies [26].

Presence of supernumerary teeth belongs to pathological condition [27]. They have been found in the antemolar space in several mouse mutant strains, for example, in transgenic mice with overexpressed *ectodysplasin* (*Eda*) or its receptor (*Edar*) [28,29], or in *ectodin*-deficient mice [30]. Similarly, a supernumerary tooth in front of molars also develops in *Spry*2 or *Spry*4 deficient mice [13,31].

In the present study, the fusion between the *Shh* expressing domains R2 and M1 was standardly apparent in control genotype and in the mice with a higher dosages of *Spry* genes (Figure 1A; Figure 2) where the supernumerary tooth forms only very rarely. In contrast, the genotypes with lower *Spry* gene dosages, did not exhibit the fusion of R2 and M1 *Shh* signaling domains. (Figure 1B; Figure 2). The lack of fusion resulted in supernumerary tooth primordium formation in majority of embryos according to genotype. The R2 *Shh* signaling domain stayed separate anteriorly to the persisting original early M1 *Shh* domain and the R2 bud developed progressively into a supernumerary tooth primordium (Figure 1B). This provides clear evidence that in mutant mice with an extra tooth in front of M1, the R2 *Shh* signaling domain indicates a signaling center of separately evolving supernumerary tooth. The R2 rudiment in such cases develops autonomously giving rise to the supernumerary tooth primordium, instead of being incorporated into the M1.

This phenomenon was confirmed by Cre-loxP technology allowing the tracing of the cells originally expressing *Shh* in two separate domains in R2 and M1primordia that are finally localized in one area of M1 germ at E16.5 (Figure 4A, B). In contrast, in *Spry*4 deficient embryos, a presence of two separate areas of cells originally expressing *Shh* in a supernumerary tooth germ and in M1 germ documented the non-fusion of R2 and M1 *Shh* expression domains at E14.5 with a consequent supernumerary tooth formation anteriorly to M1 primordium (Figure 4C, D).

In the present study, the number of the supernumerary tooth germs decreased in developmentally more advanced specimens (higher body weights). Regression of the supernumerary tooth primordium has been previously also reported in *Spry*4−/− and *Spry*2−/− mice. The presence of supernumerary tooth germ during prenatal development has been detected to be significantly higher than the presence of supernumerary tooth in adults *Sprouty* deficient mice [31].

In general, the supernumerary teeth were more frequent with decreasing dosages of *Spry2*;*Spry4*, which demonstrates essential roles of *Sprouty* genes for normal tooth development and patterning.

### *Sprouty* gene dosages

The influence of dose of mutant genes on mouse phenotype has been confirmed previously. Variation of *Pax9* mutant alleles causes oligodontia, hypoplastic or missing lower incisors and third molars in mouse [32]. Palate development is sensitive to *Spry2* dose [33]. Using *Spry* mutant mice, the loss of function in *Spry* genes results in the increased number of incisors in the upper jaw [19].

Our data document that the presence of rudimentary and functional tooth germs is not totally disrupted by lacking *Spry* genes. However, the dosages of *Sprouty2* and *Sprouty4* genes affect the timing and formation of the early tooth primordia in the cheek region of mouse mandible. The consequent appearance of *Shh* expression domains in three distinct areas corresponded to those observed in controls, but the expression was slightly prolonged. The prolongation was followed by a delay (later beginning) of the *Shh* expressions in R2 and M1 areas compared to control genotype. Interestingly, the prolongation of the *Shh* expression was more obvious with the decreasing dosage of *Spry2* gene (see Figure 2B). This suggests that *Spry2* has a higher impact in tooth development than *Spry4.*

### Molecular regulation of supernumerary tooth primordium formation

*Sprouty* genes belong to evolutionarily conserved family and they are essential for the normal development of craniofacial structures including dentition. They encode a negative regulator of FGF and other RTK (Receptor Tyrosine Kinases) signaling and indirectly influence the expression of *Shh* [34-36].

It has been reported that mutations in *Spry*2 or *Spry*4 genes cause the formation of a supernumerary tooth in mouse diastema through increased FGF signaling [13,37]. FGF signaling pathway is active in the epithelium and mesenchyme and plays a role in the stimulation of cell proliferation [6,38]. It also prevents apoptosis [8]. This is in agreement with findings that after loss of function of the *Sprouty* genes and increased FGF signaling [34,39] the apoptosis is reduced and cell proliferation is increased in the area of dental rudiments MS and R2 [37]. Such rudiment “revitalization” results in the formation of the supernumerary tooth primordium [31,37]. Several genes from FGF family induce *Shh* expression, which then affects other members of FGF family [13,34,40,41]. This feedback model was confirmed in supernumerary tooth in *Spry*2 deficient mice [13] and also in the limb buds development in mice [42]. According to this the increased *FGF* expression could prolong the *Shh* expression.

### Developmental arrest of the supernumerary tooth development in *Spry* mutant mice

Frequency of the supernumerary tooth presence in the erupted dentition has been detected to be substantially lower than prenatal presence of supernumerary tooth primordium [31]. Based on our results we tried to explain this phenomenon and outline hypothetically the signaling pathways that could stop supernumerary primordium development in *Spry2*;*Spry4* mutant mice during later prenatal period.

**Figure 5:**
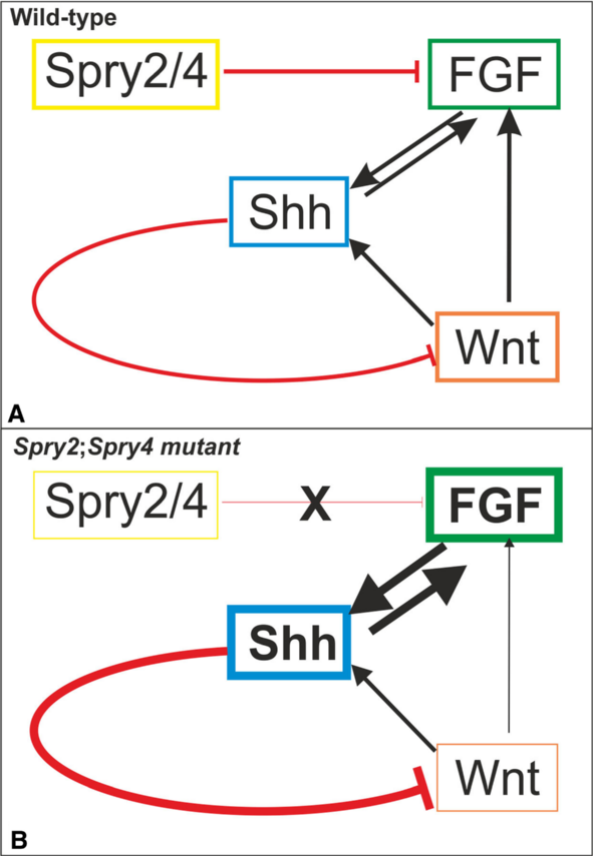
A tentative model of molecular control of formation and inhibition of supernumerary tooth development in mouse lower diastema in *Spry2*;*Spry4* mutants. In wild-type mouse embryos **(A)**, Spry2/Spry4 antagonizes Fgf signaling [13] and thus indirectly influences Shh level [13,31,39]. Shh is a negative regulator of Wnt [18] and plays a role in tooth separation [43]. In Spry2;Spry4 mutants **(B)** loss of function of the *Sprouty* genes leads to increasing of FGF signaling [13,34,39]. This results in a reduction of apoptosis and stimulation of cell proliferation in the MS and R2 rudiments, which results in the formation of the supernumerary tooth primordium [31,37]. Higher level of Fgf causes a higher level of *Shh,* which helps R2 rudiment to separate from M1 tooth germ. However, the elevated Shh strongly inhibits the Wnt signaling, decreased Wnt signaling cannot prevent an independent development of R2 rudiment as supernumerary tooth primordium, which finally regresses. The thickness of lines and frames symbolizes approximate levels of signaling activity.

Interaction between Shh and Wnt signaling has been proven during tooth development, where Shh acts as a negative feedback regulator of Wnt in diastemal tooth development [18]. *Wise*-null mice have shown that reduction of Shh activity leads to continuous R2 development by increased Wnt signaling [18]. Using feedback model [13,39] we can propose that the Shh signaling is strongly upregulated by an increased FGF signaling in *Spry2*;*Spry4* double-knockout mice. It is known that Shh is required for separation of teeth [43] by antagonizing Wnt signaling [18]. Using these knowledge we suggest a model explaining why supernumerary tooth primordia ceased to be detected at later stages in some genotypes (definitely in *Spry*2+/+;*Spry*4−/−, *Spry*2+/−;*Spry*4−/−) in diastema (Figure 5). The maintenance of Shh signaling is involved in the separation of R2 rudiment from the developing first molar and indicates formation of supernumerary tooth primordium (Figure 5B). Higher level of Shh should strongly antagonize Wnt signaling, which is necessary for proper tooth development. Decreased levels of Wnt might stop continuous R2 development and the primordium of supernumerary tooth regress. This would mean that Shh modulates the levels of Wnt signaling during tooth development (Figure 5B). However, the question remains, why some tooth primordia still develop into erupted supernumerary teeth [13,31].

The present data document that changes of *Spry*2 and *Spry*4 gene dosages have consequences for developmental dynamics and patterning of tooth primordia and that increasing *Spry*2 and *Spry*4 gene dosages allow approaching the normal tooth development in mutants.

## Conclusions

The *Shh* signaling domain of R2 rudiment transiently synchronously co-exists with early M1 *Shh* signaling domain.

These two signaling domains finally fuse together giving rise to pEK in prospective functional M1 germ between E14.5 and 14.7 (embryos with body-weight in range 270-320 mg) under physiological conditions.

By contrast, the non-fusion of original R2 and early M1 *Shh* signaling domains results in the subsequent development of the supernumerary tooth primordium on the base of R2 rudiment.

The formation of the supernumerary tooth germs depends on the dosages of *Sprouty2* and *Sprouty4* genes – the number of supernumeraries increases with decreasing *Spry*2;*Spry*4 gene dosages.

## Methods

### Mouse embryos

We used transgenic mouse strain *Spry*2ORF-null allele/*Spry*4ORF-null allele/B6.Cg-*Shh*tm1(EGFP/cre)Cjt/J (original strain of B6.Cg-*Shh*tm1(EGFP/cre)Cjt/J was crossed with *Spry*2ORF-null allele and *Spry*4ORF-null allele – kind gift of Ophir Klein), where EGFPCre fusion product [44] is inserted into the endogenous *Shh* locus. GFP fluorescence co-localizes with *Shh* mRNA [45]. Males *Spry*2ORF/*Spry*4ORF /B6.Cg-*Shh*tm1(EGFP/cre)Cjt/J were crossed with females *Spry*2ORF/ *Spry*4ORF or *Spry*2ORF or *Spry*4ORF in order to get embryos with different dosages of *Spry2* and *Spry4* genes (Table 1).

B6.129S6‐*Shh* < tm2(cre/ERT2)Cjt>/J transgenic mice carrying the gene for the fusion product of *Shh* and tamoxifen-inducible Cre recombinase were reciprocally crossed with B6.129S4-Gt(ROSA)26Sortm1LacZSor/J mice carrying a reporter gene (*LacZ*) inserted into the Gt(ROSA)26Sor locus to enzymatic detection of beta-galactosidase activity. These mouse strains allow following the fate of cells originally expressing *Shh* and their descendants.

*Spry*4ORF-null allele mice were crossed with B6.129S6‐*Shh* < tm2(cre/ERT2)Cjt>/J transgenic mice and with B6.129S4-Gt(ROSA)26Sortm1LacZSor/J mice to generate the mice carrying the phenotype of *Spry*4ORF-null allele strain (exhibiting the supernumerary tooth [13]) and allowing formation of tamoxifen-inducible Cre-loxP system by reciprocal crossing of *Spry*4ORF-null allele/B6.129S6‐*Shh* < tm2(cre/ERT2)Cjt>/J transgenic mice with *Spry*4ORF-null allele/ B6.129S4-Gt(ROSA)26Sortm1LacZSor/J mice. These strains differ from the aforementioned only by presence of non-functional copies of *Spry4* gene.

The mice were genotyped using standard protocol (Jackson Laboratory, Maine, USA). *Spry*2ORF-null allele and *Spry*4ORF-null allele mice was a kind gift of Ophir Klein, other mouse strains were purchased from Jackson Laboratory, Maine, USA.

### Harvesting of embryos

The appropriate mice were mated overnight and the midnight before the morning detection of the vaginal plug was determined as the embryonic day (E) 0.0. The embryos were harvested between E11.5 and 16.5. The pregnant mice were sacrificed by cervical dislocation and the embryos were removed from uterus. Immediately after removing of the embryos, their wet body weight was determined for refining their chronological staging. The body weight correlates very well with the developmental progress of early stages of odontogenesis [46]. In order to get a detail series of progressive tooth development and a homogeneity of data, mouse embryos were always harvested in several time horizons at each E: the embryos at E12.3; 13.3; 14.3; 15.3 and 16.3 were collected between 6 and 9 AM; embryos at E11.5; 12.5; 13.5; 14.5; 15.5 and 16.5 were collected between 9 AM and 3 PM, and the embryos at E12.7; 13.7; 14.7 and 15.7 were collected between 3 and 6 PM. A sample of tissue of each specimen was genotyped. The animals’ treatment satisfied the requirements of the Institutional Review Board of the Institute of Experimental Medicine, Academy of Sciences of the Czech Republic, Prague, Czech Republic.

### Mandible dissection

The mandibles were dissected from embryonic heads and then part of jaws comprising of tooth germs from cheek regions were micro-dissected. The specimens were ranked according to their genotype and body weight at each time horizon. Using this method, a detailed series of progressive stages of dental development was established for each individual genotype (see Table 1).

### Epithelium dissociation and fluorescent microscopy

The dissected tooth germs from the lower jaw of EGFP positive embryos were put into the Hank’s solution (Sigma Aldrich). The Hank’s solution was replaced by 1% trypsin solution (Difco Laboratories) in 4°C for one to two hours (according to the developmental stage of embryos) to dissociate the epithelium from the mesenchyme. Dissociated epithelia were documented in the Stop solution (20% FCS - Sigma Aldrich) using the inverted fluorescent microscope Leica AF6000 (Leica Microsystems GmbH, Germany). *Shh* expression domains were determined according to the green fluorescence in the cells actually expressing *Shh*.

### Evaluation of tooth development

The stage of tooth development (bud, cap and bell) was determined on the basis of morphology of dental epithelium (Figure 1). We were looking for *Shh* expression domains in dental primordia in dissociated epithelia because *Shh* is generally considered as a marker of early tooth development.

We evaluated the dynamics of *Shh* expression in the dental epithelium in all available genotypes based on the combinations of the alleles of *Spry*2 and *Spry*4 genes. The specimens of all genotypes were compared, including controls *(Spry2*+/+;*Spry4*+/+). In total, 629 samples of dental epithelia were evaluated. The tooth primordium was determined as supernumerary when there was a separate epithelial structure with own signaling center. This structure was localized in front of M1 anlage and there was evident boundary between them.

### Tamoxifen administration and X-gal staining

The dose of 9 mg of tamoxifen/40 g body weight [47] was intra-peritoneally injected in pregnant female mice at E13.5. This dose is not life-threatening for mouse females or embryos, but it is sufficient for activation of Cre-recombinase. The embryos were harvested at E15.5 and E16.5 (48 and 72 hours after tamoxifen application). The X-gal (Sigma) concentration in the staining buffer was 3 mM. Separated heads of embryos with positive staining were post-fixed in PFA (4%) overnight. The samples were washed in PBS and lower jaw was dissected and photographed using a Leica MZ6 stereomicroscope with Leica EC3 digital camera (Leica Microsystems GmbH, Wetzlar, Germany). After photo-documentation, the samples were post-fixed in Bouin solution for 2 weeks and then histologically processed.

### Histology of X‐gal stained samples

The samples were routinely embedded in paraffin and 10 μm thick sections were prepared. After paraffin removal and hydration, the sections were counterstained with Fast red (Fluka). The stained sections were dehydrated and cover slipped using Neomount (Merck).

## Abbreviations

MS: Mesial segment
R2: Tooth rudiment
M1: First molar
Spry2: Sprouty2
Spry4: Sprouty4
Shh: Sonic Hedgehog
pEK: Primary enamel knot
E: Embryonic day
EKs: Enamel knots
Lrp: Low-density lipoprotein receptor-related protein
Fgfr: Fibroblast growth factor receptor
ER: Estrogen receptor
X-gal: 5-bromo-4-chloro-3-indolyl-β-D-galactopyranoside
FGF: Fibroblast growth factor
GFP: Green fluorescent protein
